# Prevalence of HPV infection among sexually active adolescents and young adults in Brazil: The POP-Brazil Study

**DOI:** 10.1038/s41598-020-61582-2

**Published:** 2020-03-18

**Authors:** Eliana Marcia Wendland, Luisa Lina Villa, Elizabeth R. Unger, Carla Magda Domingues, Adele Schwartz Benzaken, POP-Brazil Study Group, Ana Goretti Kalume Maranhão, Natalia Luiza Kops, Marina Bessel, Juliana Caierão, Glaucia Fragoso Hohenberger, Jaqueline Horvath, Giovana Tavares, Barbara Pereira Mello, Aniusca Vieira dos Santos, Maiquidieli Dal Berto, Claudia Bica, Gerson Fernandes Mendes Pereira, Flavia Moreno

**Affiliations:** 1 Hospital Moinhos de Vento, Porto Alegre, Brazil; 2 Graduate Program in Health Sciences and Graduate Program in Pediatrics, Federal University of Health Sciences of Porto Alegre, Porto Alegre, Brazil; 3 Universidade de São Paulo and Instituto do Câncer do Estado de São Paulo (ICESP), São Paulo, Brazil; 4 Division of High-Consequence Pathogens and Pathology, National Center for Emerging and Zoonotic Diseases, Centers for Disease Control and Prevention, Atlanta, USA; 5 National Immunization Program, Ministry of Health, Brasilia, Brazil; 6 Tropical Medicine Foundation Heitor Vieira Dourado and Global Aids Healthcare Foundation, Manaus, Brazil; 7 Programa de Pós-Graduação em Ciências Farmacêuticas, Faculdade de Farmácia, Universidade Federal do Rio Grande do Sul, Porto Alegre, Brazil; 8 Graduate Program in Pathology, Federal University of Health Sciences of Porto Alegre, Rio Grande do Sul, Brazil; 9 Department of Chronic Conditions Diseases and Sexually Transmitted Infections, Ministry of Health, Brasília, DF Brazil

**Keywords:** Epidemiology, Translational research

## Abstract

For Brazil, there are no nationwide data on HPV prevalence against which the impact of the HPV immunization program can be measured in the future. Therefore, we aim to evaluate the prevalence of genital HPV infection among adolescents and young adults in Brazil. A cross-sectional, multicentric, nationwide survey was conducted between September 2016 and November 2017. Sexually active unvaccinated women and men aged 16 to 25 years old were recruited from 119 public primary care units, including all 26 state capitals and the Federal District. All participants answered a face-to-face interview and provided biological samples for genital HPV analysis. We used an automated DNA extraction method and HPV genotyping was performed using the Linear Array genotyping test (Roche). Of 7,694 participants, 53.6% (95% CI 51.4–55.8) were positive for any HPV type. The prevalence of high-risk HPV types was significantly higher in women (38.6% vs. 29.2%, *P* < 0·001). The HPV types included in the quadrivalent vaccine were detected in 1002 (14.8%) specimens, with a different pattern of HPV infection between sexes. Characteristics associated with overall HPV detection included female gender, self-declaration of race as brown/pardo, lower socioeconomic class, single or dating, current smoking and having 2 or more sex partners in the past year. We found a high prevalence of HPV, with significant differences between regions. Our data provide information that may be considered when developing HPV prevention policies and constitute a baseline against which the impact of the HPV immunization program in Brazil can be measured in future years.

## Introduction

Human papillomavirus (HPV) is globally the most common sexually transmitted infection^1^, and it is strongly associated with cervical, anogenital and oropharyngeal cancers^2,3^. HPV is one of the main causes of mortality among women in underdeveloped countries^1,4,5^. Although the prevalence of HPV has already been evaluated in some specific groups and regions, there are no data on HPV prevalence in young general populations of the different regions of Brazil^6^.

The prevalence and types of circulating HPV vary widely both among different populations and among age groups within populations. All regions of the world have shown an overall decline in prevalence according to age, except Latin America and the Caribbean, where the prevalence increases later in life, presenting a bi-modal distribution^1^. In addition, the prevalence and type-specific HPV frequency can change according to race, with a higher incidence in indigenous^7^ and black populations^8^.

The introduction of HPV vaccination is an opportunity to prevent infection and associated lesions, thus changing the patterns of mortality by cervical cancer. Brazil introduced an HPV immunization program using a quadrivalent vaccine in 2014 for children 9 to 14 years old and is currently adopting a 2-dose vaccination schedule (0–6 months)^9^. Although vaccine efficacy in decrease cervical intraepithelial neoplasia has been demonstrated in clinical trials^10–12^, measuring the impact of vaccination on HPV prevalence in young populations is important to evaluate the effectiveness of immunization against HPV in different scenarios under pressure of the vaccines. The impact of national HPV vaccination programs is being evaluated with different strategies, including data obtained from residual cervical screening^13–18^, including women 25 years or older, that are dependent on screening rates and the population profile of each specific country. Few studies have had the opportunity to use data from national surveys^19^ and evaluate young populations.

This study reports prevalence data on type-specific genital HPV in a nationwide sample of adolescents and young adults who use the Public Health System in Brazil. These data allow comparison between different geographical regions and provide important baseline estimates to evaluate the impact of the HPV vaccination program.

## Methods

### Study design and population

In this cross-sectional study, we evaluated the prevalence of HPV types in a convenience sample of sexually active women and men, aged 16 to 25 years from public primary health care units of the 26 state capitals and the Federal District of Brazil, from September 2016 to November 2017. Participants were recruited through different approaches as individuals who came to the unit for any reason, community health agents extended personal invitations, and nurses in school-based health promotion programs. The study sample of 7935 was calculated to detect a prevalence of at least 30% with an 80% power and to detect a difference of at least 5.2% between the regions of the country. Males were included (M:F ratio of 1:6) to allow subsequent assessment of herd immunity as a result of the HPV vaccination program in future years. This research was approved by the Moinhos de Vento Hospital human subjects research board, and written informed consent was obtained from all participants (Approval No. 1607032). We excluded pregnant women, those who had undergone hysterectomy or trachelectomy, and participants who had ever had cervical intraepithelial neoplasia grade 2 or higher. The study protocol was published previously^20^.

### Data collection

All participants were interviewed by primary care professionals trained specifically for the study using structured questionnaires on risk factors for HPV infection and demographic and behavioral data. Socioeconomic status distribution is a composite score calculated based on the number of household assets, degree of education of the household head and presence of monthly paid housekeeper^21^. Race/skin color was self-reported. We also asked about current and past cigarette smoking, educational level, marital status, number of sex partners in the last five years and last 12 months, age at first sexual intercourse, and condom use.

For women, the cervical sample was obtained using the Qiagen HC2 DNA collection device according to the manufacturer’s instructions. Men were instructed to self-collect samples from the entire surface of the scrotum, glans penis/coronal sulcus and penile shaft using a saline-wetted Dacron swab, under supervision of a health professional. The cervical and genital swabs were placed in a tube containing 1 mL of Digene Specimen Transport Medium (STM), stored at controlled room temperature (15–25 °C) and shipped to a central laboratory weekly where the samples were aliquoted and stored at −80 °C until processing.

### HPV genotyping test

DNA was extracted from 0.5 mL of specimen transport medium (STM) using magnetic beads for isolation and purification on a robotic system (MagNA Pure LC 2.0; Roche) according to the manufacturer’s extraction instructions and the DNA concentration in the extract was determined using the NanoDrop 2000 (Thermo Scientific™).

HPV genotyping was performed using the Roche Linear Array® (LA) genotyping test which amplifies a 450 bp fragment in the L1 gene. Per reaction, 25 μl working master mix was combined with DNA extract (between 100–150 ng) diluted in 25 μl of ultrapure DNase/RNase-Free water. Polymerase chain reaction cycling conditions and hybridization were performed as recommended by the manufacturer. Probes could detect 37 types of HPV simultaneously^22^. The assay incorporated β-globin as an internal control for sample amplification. To ensure reproducibility of LA, an automated AutoBlot instrument (Fujirebio) was used for the hybridization and wash steps^23^. As the HPV 52 probe cross-reacts with types 33, 35 and 58, additional analyses were performed, if needed, using a type-specific real-time PCR assay^24,25^. Samples negative for β-globin and HPV (n = 312, 14.68% in men; n = 4, 0.07% in women) were considered inadequate and excluded from analysis. Six hundred eighty-seven (8.93%) men and women were considered nonresponders because they used an incorrect sampling technique or sample DNA was less than 5 ng/µl. We chose not to genotype these samples to avoid potential bias in the results. From the analyzed samples, eight (0.13%) were considered inadequate after genotyping.

HPV results were grouped as follows: positive for any HPV type, positive for 13 high-risk HPV (16, 18, 31, 33, 35, 39, 45, 51, 52, 56, 58, 59, 68), positive for other HPV (6, 11, 26, 40, 42, 53, 54, 55, 61, 62, 64, 66, 67, 69, 70, 71, 72, 73, 81, 82 [IS39], 83, 84, 89 [CP6108]), positive for vaccine types (quadrivalent: 6, 11, 16, 18; nonavalent: 6, 11, 16, 18, 31, 33, 45, 52, 58), and multiple types (more than one HPV type).

### Statistical analysis

The variable number of partners in the past year was categorized as ≤1 or >1 and the variable number of sexual partners in the last five years was categorized as ≤1, 2–3 and ≥4, according to the variable distribution. Social class was categorized according with the Associação Brasileira de Empresas de Pesquisa (ABEP – Brazilian Association of Survey Companies b) who uses a system based on the purchasing power and the level of education of the families to identify social and economic status, ranging from the highest (Class A) to the lowest (Class E)^26^.

We used the response rate definition from the American Association for Public Opinion Research, which defines the response rate as the number of participants divided by the number of persons that were ever contacted and screened as eligible^27^.

Sampling weights were applied to the data from our convenience sample in each capital to estimate HPV prevalence among the male and female population aged 16–25 years according to the Brazilian Census^28^. SAS software (Statistical Analysis System, SAS Institute Inc., Cary, N.C.), version 9·4, was used to conduct the analyses, and a 2-sided confidence level of 5% was determined to be statistically significant. Data with a normal distribution (Anderson-Darling test) were analyzed with Student’s t test or ANOVA, and the Chi square test was used for categorical variables.

The association between demographic and behavioral characteristics and the prevalence of HPV (overall and HR-HPV) was evaluated through Poisson regression with robust variance for binary outcomes. Variables with *P* < 0.20 in univariate analysis were included hierarchically (sociodemographic, socioeconomic, and behavioral characteristics, consecutively) in a multivariate model. The multivariate model was controlled by other variables included in the model, using the Hosmer-Lemeshow procedure^29^.

For each region, a univariate model was used to evaluate the prevalence ratio of characteristics in relation to HPV. Additionally, a multivariate model (adjusted for all included variables) was used to evaluate the prevalence ratio among each of the five regions of Brazil, using the South region as reference (region with lowest HPV prevalence). Interaction terms between variables were examined to evaluate effect modification. The vaccine status was investigated, and 810 participants who reported having been vaccinated were excluded from the analysis.

### Patient and public involvement

No patients were involved in setting the research question or the outcome measures, nor were they involved in developing plans for recruitment, design, or implementation of the study. No patients were asked to advise on interpretation or writing up of results. The results from the present study will be disseminated through institutional websites, written communication, events and conferences, networks, and social media.

### Ethical approval

All procedures performed in studies involving human participants were in accordance with the ethical standards of the Moinhos de Vento Hospital research board (Approval No. 1607032) and with the 1964 Helsinki declaration and its later amendments or comparable ethical standards.

## Results

### Characteristics of participants

Of the 7694 recruited participants, 6388 (83.0%) provided valid samples for HPV analysis (5268 women and 1120 men). There were no significant differences in race/skin color, educational level or family income in the population that was able to provide a valid sample (Supplement 1). Higher social class, younger age and the Southeast region had higher levels of inadequate sample (Supplement 1). The overall response rate^27^, defined as the number of eligible people who agreed to participate in the study, was 99.1%.

The majority declared themselves as brown/pardo (n = 3671/ 57.0%), 3817 were not engaged in a formal relationship (63.5%), 3402 were socioeconomic status C (55.5%), and 3571 were attending or had finished secondary school (55.9%) (Table 1). Although half used condoms at some point during their lives, less than 40% reported condom use in the last intercourse encounter. The mean age at first sexual intercourse was 15.3 years (95% CI 15.2 to 15.4), and the majority (79.0%) of women had only one partner in the past year. The age of first intercourse was lower in men (14.9 (95% CI 14.8 to 15.1)) than in women (15.5 (95% CI 15.4 to 15.5)), and men had more partners in the last year (44.6%) than women (20.9%).

**Table 1:** Sociodemographic and Sexual Behavior Characteristics of the Study Population. POP-Brazil Study, 2017.

	Number	% (95% CI)
Sex		
Female	5268	63.6 (61.2–66.0)
Male	1120	36.4 (34.0–38.8)
Age, y		
16–21	3422	53.3 (51.1–55.5)
22–25	2966	46.7 (44.5–48.9)
Race/color^a^		
White	1519	23.3 (21.4–25.2)
Black	1013	17.5 (15.8–19.2)
Brown/pardo	3671	57.0 (54.8–59.1)
Other	151	2.2 (1.6–2.8)
Marital status		
Single	1339	22.6 (20.6–24.6)
Dating	2403	39.9 (37.7–42.0)
Married/living with partner	2570	36.5 (34.5–38.5)
Widowed/divorced/separated	75	1.0 (0.6–1.2)
Socioeconomic status^b^		
A	97	1.7 (1.0–2.3)
B	991	14.8 (13.3–16.4)
C	3402	55.5 (53.3–57.6)
D-E	1898	28.0 (26.1–29.8)
Education level^a^		
Elementary school to complete or not	1373	23.4 (21.5–25.2)
Secondary school to complete or not	3571	55.9 (53.7–58.1)
Graduate to complete or not	1443	20.7 (19.0–22.4)
Regular condom use	3190	50.0 (47.8–52.2)
Condom use in the last intercourse	2409	37.6 (35.5–39.7)
No. sex partners in the past year^a^		
<2	4509	69.8 (67.6–72.0)
≥2	1636	30.2 (28.0–32.3)
No. sex partners in the past 5 years^a^		
<1	1868	29.8 (27.7–31.7)
2–3	1926	33.3 (31.2–35.4)
≥4	1834	36.9 (34.7–39.2)
Age at first sexual intercourse	7473	15.21 (15.1–15.3)

^a^Does not sum to 6388 because some of the responses are missing; ^b^The social class distribution is a composite score calculated based on the number of household assets, degree of education of the household head and presence of monthly paid housekeeper.

### HPV prevalence and genotype

**Figure 1:**
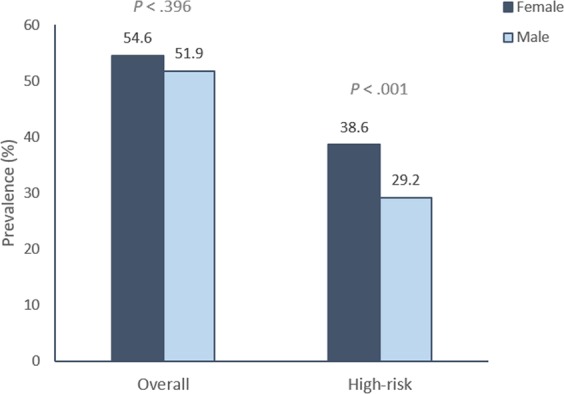
Prevalence of Any and High-Risk Human Papillomavirus (HPV) According to Sex. POP-Brazil Study, 2017. High-risk HPV types are defined as HPV types 16, 18, 31, 33, 35, 39, 45, 51, 52, 56, 58, 59, and 68.

From examined samples, 3447 were positive for any HPV type (53.6%; 95%CI 51.4 to 55.8), with no differences between women (2851, 54.6%; 95%CI 52.5 to 56.6) and men (596, 51.8%; 95%CI 47.0 to 56.7). When stratified by age group (16–21 and 22–25), there was a significant difference only among women (*P* < 0.001), with a higher prevalence of HPV infection in the 16–21 year old group (1652, 57.9%; 95%CI 58.8 to 64.2) than in the 22–25 year old group (1199, 42.1%; 95%CI 44.2 to 50.2). Detection of both overall HPV and HR-HPV prevalence varied by age, being highest among 18–19-year-olds and decreasing in older ages. The prevalence of HR-HPV types was 35.2% (2358; 95%CI 33.1 to 37.2), with significant differences between sexes, with a higher frequency in women (2030, 38.6%) than men (328, 29.2%) (Fig. 1).

Quadrivalent HPV types were detected in 1002 (14.8%) specimens [27.9% (95%CI 25.5 to 30.4) of those with any HPV detected], with no significant differences between men and women (data not shown). Nonavalent HPV types were detected in 1842 (27.7%; 95%CI 25.8 to 29.6) specimens being 51.6% (95%CI 48.7 to 54.6) of all HPV positive samples.

Although most individuals who tested positive for HPV had only one HPV type (1411, 42.2%; 95%CI 39.3 to 45.1), multiple types were detected in 2036 (31.0%; 95%CI 29.0 to 32.9) participants: 883 (26.7%; 95%CI 24.1 to 29.3) had 2 types, 484 (12.2%; 95%CI 10.5 to 14.0) had 3 types, and 669 (18.8%; 95%CI 16.6 to 21.1) had 4 types or more. There was a statistically significant difference in the proportion of multiple types between sexes (33.0% in women and 27.5% in men, *P* < 0.02).

**Figure 2:**
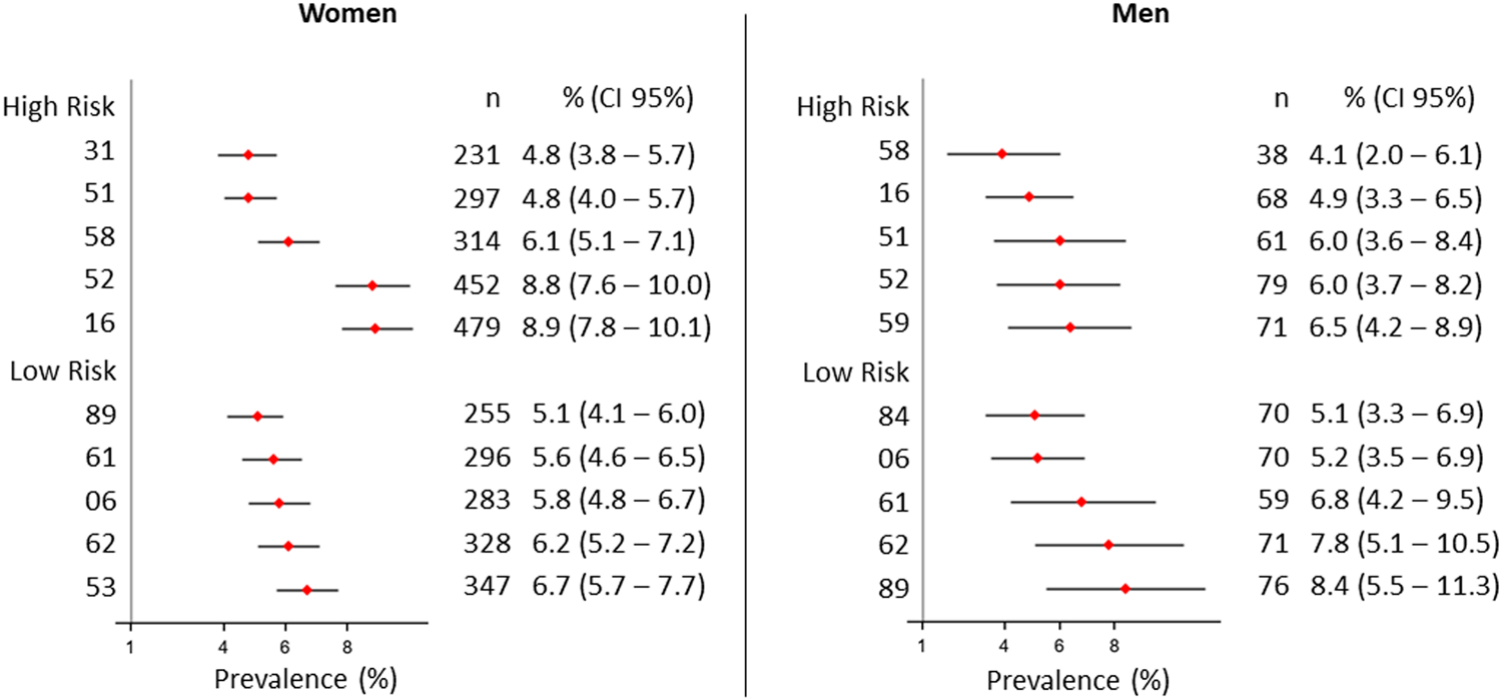
Most Frequent High- and Low-Risk HPV Types in Men and Women in the POP-Brazil Study. The lines indicate 95% confidence intervals.

The most prevalent HPV types were HPV 52 (531, 7.8%; 95%CI 6.7 to 8.9), HPV 16 (547, 7.5%; 95%CI 6.5 to 8.4), HPV 62 (399, 6.8%; 95%CI 5.6 to 7.9), HPV 89 (331, 6.3%; 95%CI 5.0 to 7.5), and HPV 61 (355, 6.0%; 95%CI 4.9 to 7.2). Overall, 247 (3.8%; 95%CI 2.9 to 4.6) participants were positive for HPV 18, and 757 (10.73%; 95%CI 9.5 to 11.9) were positive for both HPV 16 and 18. The prevalence of HPV 6 was 5.6% (353; 95%CI 4.7 to 6.4), and 74 participants (1.4%; 95%CI 0.9 to 1.9) were positive for HPV 11. The pattern of HPV infection differed widely between sexes. While HPV 16 (479, 8.9%; 95%CI 7.7 to 10.1) and 52 (452, 8.8%; 95%CI 7.6 to 10.0) were the most prevalent HR-HPV types in women, HPV 59 (71, 6.5%; 95%CI 4.2 to 8.9) and 52 (79, 6.0%; 95%CI 3.7 to 8.2) were the most frequent in men (Fig. 2).

### Determinants of HPV detection

Detection of any HPV detection was associated with female gender, brown/pardo color, socioeconomic status D-E, being single or dating, current smoking and having two or more partners in the past year. Associations for detection of HPV-HR types were similar to those for detection of any HPV types, except brown/pardo color and former smoker were not significant for HPV-HR types (Table 2).

**Table 2:** Characteristics Associated with any type and High-Risk Genital HPV Prevalence: a Multivariate Model. POP-Brazil Study, 2017.

	Any HPV type	High-risk HPV types
	Adjusted prevalence ratio (95% CI)	
Sex		
Female	1	1
Male	0.85 (0.75–0.95)	0.62 (0.52–0.75)
Age, y		
16–21	1	1
22–25	0.92 (0.84–1.00)	0.88 (0.78–1.01)
Race/color		
White	1	1
Black	1.03 (0.90–1.19)	0.99 (0.82–1.21)
Brown/pardo	1.12 (1.01–1.25)	1.08 (0.93–1.27)
Other	0.97 (0.74–1.29)	0.98 (0.69–1.39)
Socioeconomic status^a^		
A	1.19 (0.82–1.73)	1.40 (0.79–2.49)
B	1	1
C	1.08 (0.95–1.24)	1.21 (0.99–1.47)
D-E	1.15 (1.00–1.33)	1.30 (1.05–1.60)
Marital status		
Single	1.22 (1.09–1.38)	1.33 (1.13–1.58)
Dating	1.20 (1.08–1.33)	1.31 (1.14–1.51)
Married/living with partner	1	1
Widowed/divorced/separated	0.96 (0.68–1.34)	1.09 (0.70–1.70)
Smoking		
Never smoked	1	1
Current smoker	1.21 (1.07–1.38)	1.23 (1.02–1.47)
Former smoker	1.12 (1.01–1.24)	1.15 (0.99–1.33)
No. sex partners in the past year		
<2	1	1
≥2	1.24 (1.13–1.37)	1.32 (1.16–1.50)
Age at first sexual intercourse	1.00 (0.98–1.03)	0.99 (0.95–1.02)

Abbreviations: HPV, human papillomavirus. Adjusted for all variables in the table. The social class distribution is a composite score calculated based on the number of household assets, degree of education of the household head and presence of monthly paid housekeeper.

### Regional differences in HPV detection

The prevalence of any HPV differed significantly between regions with higher detection in the Northeast (919, 58.1%; 95%CI 54.8 to 61.3), followed by the Midwest (654, 56.7%; 95%CI 51.9 to 61.5), North (749, 53.6%; 95%CI 49.5 to 57.6), Southeast (617, 49.9%; 95%CI 45.3 to 54.5), and South (508, 49.7%; 95%CI 45.0 to 54.3).

The geographical regions also varied in the socio-behavioral characteristics that associated with HPV detection (Table 3). Dating increased the rate of HPV detection in the Southeast, Midwest, and Northeast regions. Current smoking and being a former smoker were associated with HPV prevalence in the Midwest, Northeast and North, and the number of sexual partners in the past year was associated with HPV prevalence in all regions except the South. Only in this region was socioeconomic status A associated with HPV infection. When we adjusted a model for sex, race/skin-color, marital status, social class, smoke, condom use, age at first intercourse and number of partners in the last year, Northeast (1.17; 95% CI 1.03–1.34) and Midwest (1.16; 95% CI 1.01–1.34) haver significantly higher prevalences when comparing to South region.

**Table 3:** Characteristics Associated with HPV Prevalence in Each Geographic Region of Brazil. POP-Brazil Study, 2017.

	Geographical region (Prevalence Ratio 95% CI)				
	South (n = 978)	Southeast (n = 1151)	Midwest (n = 1214)	Northeast (n = 1669)	North (n = 1376)
Sex					
Female	1	1	1	1	1
Male	0.94 (0.74–1.20)	0.80 (0.62–1.02)	0.97 (0.82–1.16)	1.07 (0.94–1.23)	1.13 (0.94–1.34)
Age, y					
16–21	1	1	1	1	1
22–25	1.05 (0.87–1.26)	0.86 (0.71–1.04)	0.84 (0.71–1.00)	0.91 (0.81–1.02)	0.88 (0.76–1.03)
Race/color					
White	1	1	1	1	1
Black	1.25 (0.96–1.62)	0.92 (0.69–1.24)	1.08 (0.77–1.52)	1.08 (0.89–1.30)	1.04 (0.73–1.49)
Brown/pardo	1.15 (0.91–1.44)	1.07 (0.85–1.34)	1.16 (0.92–1.46)	1.12 (0.96–1.31)	0.99 (0.75–1.31)
Other	0.99 (0.46–2.13)	1.97 (0.55–1.71)	1.22 (0.78–1.89)	1.01 (0.64–1.60)	0.79 (0.41–1.52)
Marital status					
Single	1.20 (0.93–1.55)	1.35 (1.05–1.74)	1.14 (0.91–1.43)	1.31 (1.12–1.52)	1.10 (0.90–1.35)
Dating	1.15 (0.93–1.43)	1.29 (1.04–1.61)	1.28 (1.05–1.57)	1.24 (1.09–1.41)	1.16 (0.98–1.39)
Married/living with partner	1	1	1	1	1
Widowed/divorced/ separated	0.82 (0.27–2.48)	0.88 (0.40–1.95)	0.51 (0.16–1.59)	1.49 (1.14–1.95)	1.29 (0.83–1.99)
Socioeconomic status^a^					
A	1.58 (1.05–2.38)	1.55 (0.68–3.53)	0.80 (0.46–1.39)	1.16 (0.72–1.86)	0.75 (0.23–2.38)
B	1	1	1	1	1
C	1.23 (0.98–1.56)	1.25 (0.89–1.75)	0.95 (0.78–1.16)	0.96 (0.78–1.20)	0.91 (0.71–1.16)
D-E	1.21 (0.85–1.72)	1.14 (0.79–1.65)	0.89 (0.69–1.15)	1.08 (0.87–1.34)	1.02 (0.80–1.30)
Education level					
Elementary school to complete or not	1.15 (0.86–1.53)	0.92 (0.69–1.22)	0.99 (0.80–1.24)	1.26 (1.05–1.51)	1.08 (0.82–1.39)
Secondary school to complete or not	1.13 (0.91–1.40)	0.91 (0.71–1.16)	0.84 (0.69–1.02)	1.06 (0.89–1.27)	1.26 (1.02–1.56)
Graduate to complete or not	1	1	1	1	1
Smoking					
Never smoked	1	1	1	1	1
Current smoker	1.10 (0.87–1.39)	1.16 (0.91–1.49)	1.36 (1.12–1.65)	1.34 (1.15–1.55)	1.50 (1.24–1.82)
Former smoker	0.91 (0.71–1.17)	1.00 (0.77–1.31)	1.24 (1.02–1.52)	1.20 (1.05–1.37)	1.32 (1.11–1.58)
Condom use during life	0.99 (0.82–1.20)	0.88 (0.73–1.05)	0.88 (0.74–1.04)	1.09 (0.98–1.22)	1.00 (0.86–1.16)
Age at first sexual intercourse	0.97 (0.93–1.02)	0.99 (0.95–1.04)	1.03 (0.99–1.07)	0.97 (0.94–1.00)	1.00 (0.96–1.04)
No. sex partners in the past year					
<2	1	1	1	1	1
≥2	1.21 (0.99–1.49)	1.24 (1.01–1.52)	1.32 (1.11–1.56)	1.39 (1.23–1.57)	1.23 (1.04–1.46)

Abbreviations: HPV, human papillomavirus. The social class distribution is a composite score calculated based on the number of household assets, degree of education of the household head and presence of monthly paid housekeeper. For each region, a univariate model was used to evaluate the prevalence ratio of characteristics in relation to HPV.

## Discussion

This study with over 6000 participants is the first to provide nationwide data on genital HPV prevalence in Brazil. Furthermore, to our knowledge, this is one of only a few large studies enrolling women and men simultaneously, allowing a direct comparison of HPV rates between genders. We found a high prevalence of any HPV (53.5%) and HR-HPV (35.2%) in a nationwide unvaccinated sample of 16–25-year-olds. While the overall HPV prevalence is similar between sexes, women have higher rates of HR-HPV and multiple infection. The type-specific detection of HPV infection varied widely between sexes. The geographical regions of Brazil differed in socio-behavioral characteristics that were associated with HPV detection.

Approximately half of the studied women had HPV detected. Previous small studies in the Brazilian population have shown HPV rates from as low as 6.7%^30^ up to 62.3%^31^, depending on the population evaluated and method of detection. Compared to global rates^32^, the prevalence in this study is higher than the ones reported for the same age range in South America (18.1%)^6^ and similar to the US HPV detection rate (53.8%)^18^ for 20–24 year old.

The majority of studies on HPV prevalence are focused on sex-specific populations, preventing a direct comparison between genders. In the present study, the prevalence of overall genital HPV in men was similar to women and higher than previous Brazilian studies^33^ except for the HIM Study (men aged 18–70 years), which found higher rates of HPV (72.3%)^34^. In the United States (NHANES) and England (Natsal-3) surveys have also compared the prevalence of HPV between sexes. Although the overall prevalence of HPV was higher among men in the US, this varied by age, with younger women (age 14–24 years) having higher prevalence of any type and HR-HPV^35^, similar to our results. Although we used similar genotyping methods, there were differences compared to our study. In the United States, the number of sexual partners was associated with an increase in HR-HPV prevalence in men^35^. In contrast, our study found that the number of partners increased the HR-HPV prevalence in women (from 1.6, 95%CI 1.1 to 2.1 to 1.7; 95%CI 1.2 to 2.3) but only in those who initiated sexual activity after age 15 (data not shown).

Although men and women share the main HPV types, the prevalence varies between sexes. In men, the most prevalent HR type was HPV 59, an oncogenic type related to HPV 18^36^. In women, the most prevalent HR type was HPV 16. These data emphasize the importance of screening considering that approximately 68.2% of invasive cervical cancers are attributed to HPV 16 or 18^32^.

Brazil covers a large portion of the South American continent and has high socioeconomic and cultural diversity. Therefore, it was reasonable to assume that the Brazilian population might have different risk factors associated with HPV infection from region to region. The majority of studies available were performed in women in the Southeast region and no reports were found that included all geographic regions of Brazil^33^. Although there are differences in prevalence according to region, race/color, social class and educational level were generally not significantly associated with HPV detection. Similar results have been reported in previous research studies in American and British populations^37,38^. Studies showing an effect of race and social class on HPV prevalence are often not comparable due to differences in the included population^39–41^, which is generally limited to specific groups. Behavioral characteristics, such as smoking and number of partners, may explain differences in prevalence rates according to region. Current and prior cigarette smoking was associated with HPV prevalence in all but the South and Southeast regions, possibly due to the higher prevalence of smokers in these regions (20.2% and 16.6%, respectively). Cigarette smoking contributes to cervical cancer^32^, and it has been associated with HPV prevalence^42,43^, incidence^44^, and persistence^45^.

This study has some limitations that must be noted. This multicentric community-based study was not population-based; instead, it was performed in convenience primary health care units spread throughout Brazil. We were able to weight our estimates for the age structure, but all people included in the survey were sexually active and therefore likely to overestimate the true population prevalence. Furthermore, we do not have data to weight for differences in sexual behavior between people living in capitals and other regions of the state, adding uncertainty around estimates extrapolated to the wider population. Although we have a large sample size, when we presented the prevalence stratified by sex or region, some estimates may be unstable due to the small sample size in specific categories. Additionally, males had a higher rate of invalid samples because they had lower levels of extracted DNA, similar to other studies^46^.

In summary, we report a high prevalence of any HPV type and HR-HPV types in a nationwide sample of men and women aged 16–25 years who were unvaccinated for HPV, with a higher prevalence of HR-HPV types observed in women. There were some differences in HPV prevalence across regions in Brazil that might be partially explained by behavioral variables. Our data provide information to be used in HPV prevention policies and constitute a baseline against which the impact of the HPV immunization program in Brazil can be measured in future years.

## Supplementary information

Supplement 1.
