# Supplementary material for: Prevalence of HPV infection among sexually active adolescents and young adults in Brazil: The POP-Brazil Study
Source: Sci Rep. 2020 Mar 18;10:4920. doi: 10.1038/s41598-020-61582-2 (PMC7080737; doi:10.1038/s41598-020-61582-2)
Supplement: Supplementary file 1 — Supplement 1. [file 41598_2020_61582_MOESM1_ESM.docx]

**Prevalence of HPV infection among sexually active adolescents and young adults in Brazil: The POP-Brazil Study**

**Short title:** *Prevalence of HPV in Brazil*

*Eliana Marcia Wendland^1,2^, Luisa Lina Villa^3^, Elizabeth R. Unger^4^, Carla Magda Domingues^5^, Adele Schwartz Benzaken^6^, POP-Brazil Study Group^1^

1. Hospital Moinhos de Vento, Porto Alegre, Brazil

2. Graduate Program in Health Sciences and Graduate Program in Pediatrics, Federal University of Health Sciences of Porto Alegre, Porto Alegre, Brazil

3. Universidade de São Paulo and Instituto do Câncer do Estado de São Paulo (ICESP), São Paulo, Brazil

4. Division of High-Consequence Pathogens and Pathology, National Center for Emerging and Zoonotic Diseases, Centers for Disease Control and Prevention, USA

5. National Immunization Program, Ministry of Health, Brasilia, Brazil

6. Tropical Medicine Foundation Heitor Vieira Dourado, Manaus and Aids Health Care Foundation, Manaus, Brazil and Global Aids Healthcare Foundation

**Supplement 1. Comparison between Analytic and Invalid Sample Cohorts. POP-Brazil Study, 2017**

| **Characteristic** | **Analytic sample (6388)** | **Invalid sample**  **(1306)** | ***P V*alue** |
| --- | --- | --- | --- |
| Age, y |  |  | 0.0457 |
| 16-21 | 53.3 (51.1 - 55.5) | 58.5 (53.9 - 63.0) |  |
| 22-25 | 46.7 (44.5 - 48.9) | 41.5 (37.0 - 46.1) |  |
| Race/color |  |  | 0.3179 |
| White | 23.3 (21.4 - 25.2) | 25.6 (21.5 - 29.6) |  |
| Black | 17.5 (15.9 - 19.2) | 14.8 (11.4 - 18.3) |  |
| Brown/pardo | 57.0 (54.8 - 59.2) | 56.4 (51.7 - 61.0) |  |
| Other | 2.2 (1.6 - 2.8) | 3.2 (1.2 - 5.2) |  |
| Marital status |  |  | 0.0010 |
| Single | 22.7 (20.7 - 24.6) | 25.6 (21.7 - 29.5) |  |
| Dating | 39.9 (37.7 - 42.0) | 45.5 (40.8 - 50.1) |  |
| Married/living with partner | 36.5 (34.5 - 38.5) | 28.7 (24.6 - 32.9) |  |
| Widowed/divorced/separated | 0.9 (0.6 - 1.3) | 0.2 (0.0 - 0.5) |  |
| Socioeconomic status |  |  | 0.0119 |
| A | 1.7 (1.1- 2.3) | 2.5 (0.9 - 4.0) |  |
| B | 14.8 (13.3 - 16.4) | 18.6 (15.3 - 22.1) |  |
| C | 55.5 (53.3 - 57.6) | 57.6 (53.0 - 62.1) |  |
| D-E | 28.0 (26.1 - 29.8) | 21.3 (17.4 - 25.1) |  |
| Monthly family income US$ |  |  | 0.0993 |
| < 216.00 | 21.4 (19.2 - 23.4) | 25.1 (20.9 - 29.3) |  |
| 216.00 to 431.00 | 18.9 (17.3 - 20.6) | 19.3 (15.3 - 23.3) |  |
| 432.00 to 647.00 | 41.8 (39.6 - 44.0) | 35.4 (30.8 - 40.1) |  |
| > 648 | 17.9 (16.1 - 19.7) | 20.2 (16.3 - 24.1) |  |
| Education level |  |  | 0.4716 |
| Elementary school to complete or not | 23.4 (21.5 - 25.3) | 23.8 (19.7 - 28.0) |  |
| Secondary school to complete or not | 55.9 (53.8 - 58.1) | 53.1 (48.5 - 57.8) |  |
| Graduate to complete or not | 20.7 (19.0 - 22.4) | 23.1 (19.2 - 26.9) |  |
| Geographic region |  |  | 0.0021 |
| Midwest | 13.0 (11.7 - 14.2) | 10.2 (8.4 - 12.0) |  |
| Northeast | 28.5 (26.7 - 30.3) | 26.3 (22.5 - 30.0) |  |
| North | 13.3 (12.2 - 14.4) | 10.3 (8.3 - 12.3) |  |
| Southeast | 37.9 (35.5 - 40.3) | 44.4 (39.5 - 49.3) |  |
| South | 7.3 (6.6 - 8.0) | 8.8 (7.0 - 10.6) |  |

HPV, human papillomavirus. **P* < .20; ** *P* < .05. All characteristics with *P*-values <.20 were included in the multivariate model. Exchange rate US$ = 0.26 Brazilian Real. The social class distribution is a composite score calculated based on the number of household assets, degree of education of the household head and presence of monthly paid housekeeper. Invalid samples ared due to DNA concentration equal or less than 5ng/µl.
